# Hospital and Institutional News

**Published:** 1918-09-21

**Authors:** 


### HOSPITAL AND INSTITUTIONAL NEWS.

### THE COMING HOSPITAL CONFERENCE.

If the conference to be held on Friday, October 18, at 3 p.m., at St. Thomas's Hospital, London, on the policy of the voluntary hospitals in regard to the Bill for the proposed Ministry of Health is to serve any useful purpose, hospital committees must do their own thinking before the meeting. It is easy to attend a meeting. It is difficult for most people to think. The conference, however, can form a general policy only if everyone attending it has come with definite views, considered opinions, tabulated questions, and is clear what points he wishes to have cleared up. To discover such points is not the business of the conference. To communicate the mutual confusion of the members is not its aim. If it meets in a mental fog it will only darken counsel. Therefore members have been invited to forward definite proposals—that is to say, to state what the matters are which they want to discuss. These proposals must reach the honorary secretaries of the British Hospitals Association before September 30. To this end the Council allowed the circulation of a pamphlet, the reading of which, it was hoped, would lead every member to think out his own position and that of his hospital in regard to the Bill. If the pamphlet had been less tentative, agreement or disagreement would have been easier. None the less, everyone will have formed some opinion of it, and the success of the conference can probably be measured by the number of suggestions for discussion which has reached the secretaries of the Association before September 29, the last day for sending them in.

### MEDICAL MEN IN PARLIAMENT.

It is announced that a meeting of the medical profession is to be held at the Steinway Hall, on October 1, under the chairmanship of Sir Henry Morris, to be addressed by Dr. Addison, Minister of Reconstruction, on the above subject. It is stated that the object of the meeting is "to secure the election of representative medical men to the House of Commons, so that expert advice may be available on vital questions concerning the national health." Surely, however, such expert advice could be just as well obtained from the profession when outside the Houses of Legislature. But official notices no one expects to be candid. The real value of doctors in Parliament will be to impart thereto a scientific atmosphere; and, in a less measure, of course, to assert themselves, as a rising profession is entitled to, against that predominance of legal and clerical influence on legislation which is now more and more an anachronism. It will be one of the good effects of the remodelling of medical practice that medical men may be enabled to compete with these two other learned professions in the matter of necessary leisure time to enter Parliament.

### THE SUCCESS OF THE CARDIFF APPEAL.

The recent scheme to raise £130,000 to form an endowment fund and a fund for capital expenditure in connection with King Edward VII.'s Hospital, Cardiff, has had a great success. Already the endowment fund has reached £23,000, and that for capital expenditure £72,000. More than half the total asked for has been subscribed on the snowball system, under which each friend of the hospital is asked to collect recruits of donors of 5s., and may boast of a company, a battalion, or even a brigade according to his measure of success. The work is distinctly one for all classes, and it is fair to add that the rich gave the scheme a promising start. To explain it to the prosperous war workers Mr. Rowland Evans, who speaks both Welsh and English, is ready to address any body, club, trade union, or district, and as the secretary of the Hospital Society he knows all the ramifications of the work. The waiting list is now 1,100, a blot on the city all the more scandalous from the prosperity of every citizen.

### A HOSPITAL FOR THE AMERICAN NAVY.

The Executive Committee of the London Chapter of the American Red Cross in Great Britain announces the building of a new naval hospital at a Welsh port together with convalescent quarters at an Irish naval base. This new hospital is part of the general scheme to provide institutions and canteens for the American troops along the route which they generally follow on their way through England. The principal ports are the chief centres, and the details of the hospital at Sarisbury, near Southampton, have already been given in The Hospital. The troops require one set of institutions, and the wounded another, while the headquarters of the Distributing Service, the Department of Camps and Canteens, and the principal staff of the Home Communications Service are respectively at 15 George Street, Hanover Square, and 52 Grosvenor Gardens.

### WAR EMERGENCY FORMULÆ.

The notification and suggestions of the Home Office Committee, which we deal with on p. 535, will be most welcome to the profession. The modifications and substitutes there described are being notified to the medical profession by the Committee appointed by the Home Office, August 1914, to deal with questions of economy in the use of drugs during the war. This Committee to-day consists of: Dr. J. Smith Whitaker (chairman); Sir Thomas Barlow, consulting physician, University College Hospital; Dr. A. Cox, medical secretary B.M.A.; Dr. E. Rowland Fothergill; Dr. B. A. Richmond, secretary, London Panel Committee; Dr. F. J. Smith, physician, London Hospital; Dr. W. Hale White, physician, Guy's Hospital; and the secretary, Dr. E. W. Adams, formerly of Sheffield, now medical officer of the National Health Insurance Commission.

### A SEQUEL TO THE NETLEY PROTEST.

The recent demonstration to protest against the removal by night of British wounded from 36 surgical ward at Netley Hospital, so as to make room for 100 German wounded soldiers, has been followed by the sending to the Prime Minister of a letter from the demonstrators, a letter which deserves to be recorded because it has been slurred over in the lay newspapers. After explaining that the shop stewards undertook to ascertain the truth of the rumour in order to prevent a stoppage of work, the writers add: "To our horror we ascertained a complete corroboration. As a fact, the commanding officer received instructions from headquarters at Salisbury to make room for 100 German wounded prisoners. The commanding officer assured us that every care was taken to avoid pain to our lads during removal from ward to ward. Whilst we realise that it is our humane duty to succour and mend the suffering and bleeding enemy wounded, we claim that every consideration and care should be given to our lads, and that the enemy wounded should be sent to the hospitals specially provided for them." The mixing of the wounded of the belligerent nations in the same ward in one institution has caused trouble so often that the repetition of the plan would be impossible if the military mind were not proved incapable, in administrative matters, of learning from experience.

### AN IDLE THREAT AND ITS CONSEQUENCES.

We have often pointed out that the sure signs of incompetence is the closing of a hospital's beds. This is generally admitted, except by the handful of institutions which are still endeavouring to stave off disaster by a practice which is only a thin disguise for disaster itself. Unfortunately, however, hospitals are often prepared to threaten to do that which they would never do in practice, and still endeavour to raise funds by declaring that beds must "inevitably be closed" if funds are not forthcoming. Now, idle threats are always bad policy, and it is well from time to time to remind hospitals that this particular threat is apt to cause serious trouble. A recent case is in point. In the advertisement columns of a provincial paper, an announcement recently occurred to the effect that "if the available beds" of the local hospital "were reduced by 50 per cent. it would be a great calamity." This platitude was followed by the remark that "owing to the lack of funds this seems to be inevitable." The local newspaper shortly afterwards, having taken the threat seriously, went so far as to state that a large proportion of beds had actually been closed. Events gave the statement a wide currency. The hospital indignantly denied the report, and said that it must be the product of personal malice, and further declared that if the beds had been reduced at all it would be a proof that something was seriously amiss at the hospital. The local paper may have been hasty in stating that the beds had been closed. The hospital, however, was certainly unjustified in adding that "there is no need and no intention" to reduce them. The language of its advertisement, if it meant anything at all, implied an imminent necessity; an imminent necessity is tantamount to a proposal, and between a proposal that is "inevitable" and an accomplished fact the line is necessarily fine. We are glad that a stupid advertisement has caused its authors a well-deserved amount of trouble, and we hope that the institution and other hospitals will learn from the whole incident that statements in advertisements are liable to be taken seriously, and that idle threats bring, as they deserve to bring, serious consequences.

### THE ANCIENT SHERBURN HOSPITAL.

An interesting and ancient institution is Sherburn Hospital, Durham, which is embarrassed, it is said, by an income too big for it to spend. Three miles outside Durham, the institution consists of a block of buildings which include an infirmary, a dispensary, a collection of rooms in which fifteen "in-brethren" reside, and houses for the medical officer and the master. Fifteen months ago, or thereabouts, the Charity Commissioners approved a scheme under which eight representatives of public bodies in the counties of Northumberland and Durham were added to the board. The income of the hospital is over £24,000, and the surplus on that for 1917 amounted to nearly £8,000. The invested funds which have accumulated in the course of years are understood to amount to £220,000. There seems little doubt that the pecuniary doles given to beneficiaries are small: the out-brethren and out-sisters receive from five shillings to ten shillings a week. Consequently, under cover of a proposal to extend the scope of the service rendered, it has been proposed to devote the surplus income to the benefit of other charities in the two counties. This is of course understandable, but we believe that the wishes of founders of ancient charities should be respected, and that the right line to take would be to develop the Sherburn Hospital on its historical lines both in respect of the infirmary and the house-room which it offers to old people.

### A CHANCE FOR NEW ZEALAND SECRETARIES.

With a view to stimulating the interest of officers of hospital boards in efficient methods of organisation, and with a view also to bringing out and circulating the excellent ideas which no doubt are possessed by many secretaries in regard to their functions, the Minister of Public Health of New Zealand offers a prize of £10 for the best essay on "The Economic and Efficient Management of a Public Hospital from the Secretary's Standpoint." The prize essay, together with the next in order of merit, for which a prize of £5 will be given, will be published as a pamphlet and circulated with the *Journal of the Department of Public Health*, issued under the direction of the Minister. We learn from the last issue of this journal that Mr. H. H. McCarthy, clerk to the Tauranga County Council, has been appointed secretary of the Tauranga Hospital Board. Mr. McCarthy, who was born in

Ireland in 1883 and educated at the Strand Grammar School, Whitefield, near Manchester, has been acting as secretary of the Tauranga Hospital Committee, Bay of Plenty Hospital Board, for the past three years.

### A TEST FOR SUPER-SPIES.

READERS of Mr. Laurence Binyon's recent volume on British Red Cross activities in France will remember the problems which arose at the beginning of the war on the prevention of the abuse of the Red Cross by spies and other unsatisfactory persons. A not dissimilar problem is now occupying the American Red Cross in respect of the thousands of letters now being addressed every week to persons behind the German lines. To prevent the use of a code, invisible ink, and such devices, writers must call at the local Red Cross office, of which one exists in every town in America (an office where he or she will be personally known), and write the letter there. At the Red Cross Headquarters in Washington, to which all these letters are sent, they are re-written, cut down, the sense preserved, but the wording altered. The re-written letters are then passed or rejected by a board of censors, which then dispatches them to a neutral country, where they are translated and then forwarded. No system is without loopholes, but the spy who manages to convey and receive messages in spite of these precautions would be worthy to form the hero of a scare novel.

### THE FALL OF THE CURTAIN.

THE unexpected closing of the convalescent home for wounded soldiers, known as St. Leonard's Hospital, Kincardineshire, has caused some comment because of the casualties involved in the offensive in France, and the absence of any explanation. So long as the patients, of which the institution could accommodate over fifty, came from Aberdeen, the Dundee Branch of the Red Cross was understood to be responsible, and the closing is attributed to a visit from the medical inspectors. The committee has kept, or been told to keep, its own counsel, and neither Mr. J. D. Wilkie, the secretary, nor Miss Philp, the matron, has thrown any light on an event which has come as a disagreeable shock to the inhabitants of the Stonehaven district, on whose support the hospital was accustomed to rely until extinguished in this unforeseen manner.

### THE SACRIFICE OF THE CIVILIAN.

OUR recent article under this title continues to receive justification. At Liverpool the Medical Officer of Health, in his annual report, complains of the delays in the completion of Fazakerley Sanatorium, delays extending over two years. Military assistance in extending the institution has apparently resulted in the nurses' block being allocated to female clerks and typists, while the children's quarters are used as a military boot store. Meanwhile, not only are tuberculous civilians compelled to remain at home, but consumptive soldiers invalided from the Army actually have to go back to houses scheduled as unfit for human habitation. Dr. Hope urges concerted action by the various bodies responsible for the public health, or a conference to decide between the respective claims of the military and sanitary authorities. At Leeds the same tale is heard of (in the words of the Insurance Committee), "unreasonable delay in the provision of additional accommodation for men discharged from His Majesty's Forces and civilians suffering from tuberculosis." The *Birmingham Post* describes shortage of sanatorium accommodation in Staffordshire. We foresee wholesale resignations of Insurance and Sanatorium Committee members if the Government does not make some move in this matter.

### STRIKE FEVER IN ASYLUMS.

THE conditions which produce trench fever on the Western Front have their analogues which produce what may be called "strike fever" at home, and the symptoms, if not the disease, are now making their appearance in asylums, especially in the asylums of the London area. The London Trades Council has received many lists of grievances, and the Asylums Committee of the London County Council has already been informed that, unless the demands are granted within a limited time, all the staffs will resign simultaneously. The female staffs are said to be particularly restive and implacable—a combination characteristic of their sex. The demands of the staffs probably centre in the one which proposes a general advance of 25s. a week on pre-war wages, which hitherto have been increased by some 14s. only. The food of those who live in is also a subject of grievance. The general state of affairs can be judged by the petitions which have been received by the General Purposes Committee of the Metropolitan Asylums Board. The Board's staffs ask to receive remuneration on the same scale as that adopted for the London County Council asylums' staffs. The committee has advised the managers to adopt for the non-resident staff, in respect of war bonuses, the scale contained in the award made by the Conciliation and Arbitration Board for Government Employees in respect of Civil servants with less than £500 per annum. The Government has adopted this, and the Local Government Board has offered no objection to its adoption by Boards of Guardians for their non-resident staff. The pay of the Asylums Board nurses is to be considered as soon as a scheme for the hospital nurses generally can be decided. If, as may prove the case, the conditions of the staffs in the London County Council asylums set the pace, so to speak, for asylum staffs generally, the outcome of the present negotiations will have a general importance.

### HOSPITAL DISPENSERS AND VOLUNTEER SERVICE.

THE Pharmaceutical Society, through the Pharmaceutical Central Advisory Committee, having made representations to the Local Government Board that there is need for a definite instruction to tribunals to relieve pharmacists exempted from military service of the obligation to enrol in the Volunteer Corps, the Board has now issued a memorandum relative to the matter. This states that "representations have been made to the Board that, owing to the special nature of their work, it is generally inappropriate that practising pharmaceutical chemists should be required to undertake the Volunteer obligation." This is regarded as a general instruction to tribunals, and pharmacists are advised to see that their certificates of exemption also relieve them from Volunteer service. Hospital dispensers should make a note of this, and should also bear in mind that the memorandum makes provision for the application for relief from Volunteer service of pharmacists whose certificate of exemption already granted does not provide for this. Of course, each case will be considered on its merits, but the general instruction to tribunals smooths the way.

### AN "UNBELIEVABLE" OCCURRENCE.

The members of the Southwark Board of Guardians have been horrified to hear that one of their patients, who had been in the Lambeth Infirmary, where his leg had been amputated, was discharged without crutches. Immediately on the fact becoming known, the Southwark Guardians provided the man with a crutch, and asked the Lambeth Board for an explanation. Some days elapsed, but none was forthcoming, the inference being that the clerk to the Lambeth Board of Guardians was away on holiday. A further complaint was that the sum of 18s. 9d., which the man had on his admission, was not returned to him, nor any part of it, and that in addition to being helpless and dependent on the driver of a taxi-cab, he was also penniless. As Councillor W. Savage, the chairman of the Southwark Board of Guardians, remarked, "No one could believe that any patient could be turned out of an infirmary in such a helpless manner." The explanation from the Lambeth Guardians is awaited.

### ADVERTISING FOR WORKHOUSE INMATES.

The Guardians of the New Mills Workhouse, near Wilmslow, which is now practically empty, have advertised for inmates from other Unions. The institution can accommodate nearly 200 persons, but the present full staff of officers have only twenty under their care. This is a serious matter for the officials, and the Poor-Law reformer's dream of empty workhouses is being fulfilled at Wilmslow, to the anxiety of the Guardians and their employers, for whom a new terror has been invented by the war.

### A NEW USE FOR SCHOOL GARDENS.

The children of St. Peter's School, Mansfield, have a large garden which they are cultivating under the supervision of the teachers, and one-third of it has been allotted to the Mansfield and District Hospital. All the produce grown in that portion is to go to the institution, and every Friday afternoon a hamper of vegetables is being sent there. This is an example which might be followed elsewhere.

### HERTFORDSHIRE TUBERCULOSIS REPORT.

The Medical Officer of Health has written a report on the anti-tuberculosis work in Hertfordshire for 1917. During the year a county tuberculosis hospital has been opened, with thirty beds; 719 patients were examined for the disease, and at the dispensaries, apparently twelve in number, 7,040 attendances were made; while 507 specimens of sputum were examined. The death-rate from tuberculosis had not increased over that of the previous year. There does not seem to be a clinical tuberculosis officer, perhaps owing to the war, although such is not definitely stated. In any case, the work must be much more than the Medical Officer of Health, with all the other preoccupations incidental to such a post, can manage, while the delegation of so much clinical work to local general practitioners and to nurses is undesirable as a permanent arrangement.

### VINDICATING A HEALTH VISITOR.

A report of the Kendal Health Committee states that the complaint lodged by the local doctors against the health visitor having been investigated, "no circumstances have arisen which would justify the committee in interfering with the judgment exercised by the health visitor."

### THE SHORTAGE OF PROBATIONERS.

So difficult is it to obtain volunteers for general service in certain civilian hospitals, that Miss Helen Don, honorary secretary of St. Luke's Hospital for advanced cases, in Pembridge Square, has been driven to appeal to the readers of the *Spectator* for "anyone trained or untrained, willing to offer her services," with or without a salary. She adds that the attraction of work for the military has proved overwhelming, and that civil hospitals cannot obtain probationers without the greatest difficulty except for the military side of their work. Since no lack of funds is complained of, and adequate salaries are promised, it will be interesting to see if Miss Don's appeal produces the suitable candidates which are needed at St. Luke's.

### THIS WEEK'S DRUG MARKET.

The upward movement in the price of menthol continues, and the total advance since last report is substantial; this is rather difficult to understand since arrivals from Japan have been fairly good. Japanese dementholised oil of peppermint is also considerably dearer, and the upward tendency in the price of Japanese refined camphor continues. Gum ammoniacum is very scarce, and business has been done at a very high price, liquorice juice is dearer. The advanced price of lemon oil is well maintained, and citric acid is again dearer. Aspirin continues its upward movement in price, but there are no important changes in other synthetic products. Sugar of milk is offered at lower prices, and supplies seem to be coming in more freely. Generally speaking, the market is more active and a fair all-round business is being done.

